# Advancing dye sensitized solar cell efficiency and computational insights into porphyrin based dyes

**DOI:** 10.1038/s41598-026-66026-x

**Published:** 2026-08-17

**Authors:** Noha Khamis, Yasser R. Elmarassi, Ahmed A. Hasanein, Howida A. Fetouh

**Affiliations:** Chemistry Department, Faculty of Science, Alexandria University, Alexandria, Egypt

**Keywords:** Solar cells, Porphyrin dyes, Electron injection, Light harvesting, Adsorption, Chemistry, Engineering, Materials science

## Abstract

A comparative density functional theory (DFT) and time-dependent DFT (TD-DFT) study was conducted on twelve tetraphenylporphyrin sensitizers (**I–XII**), including five free-base/zinc complex pairs and two novel asymmetric dyes (**XI**, **XII**) modeled from the benchmark **GY50** framework, to evaluate their potential for dye-sensitized solar cells (DSSCs). Electronic structures, vertical excitations, and spatial charge-transfer metrics ($\:{q}_{\mathrm{CT}}$, $\:{d}_{\mathrm{CT}}$) were evaluated at the CAM-B3LYP/LANL2DZ level using C-PCM solvation, while Multiwfn characterized wavefunction density differences. Interfacial chemisorption on anatase TiO_2_(101) was simulated using GGA/PBE-DNP on a $\:{\left({\mathrm{TiO}}_{2}\right)}_{36}\:$cluster model. For baseline dyes **I–X**, amino (-NH_2_) donor substitution reduces the HOMO–LUMO gap (E_g_) and alters orbital alignment. Target dyes **XI** and **XII** exhibit significantly narrowed HOMO–LUMO gaps $\:({E}_{g}=3.68{-}3.70\mathrm{\:eV}$in THF), intensified Q-band light-harvesting efficiency (LHE_Q_ = 0.88), enhanced charge transfer $\:{q}_{\mathrm{CT}}=0.47{-}0.48\hspace{0.17em}e$, $\:{d}_{\mathrm{CT}}=3.10{-}3.17\text{ A}^\circ$), and remarkably low internal reorganization energies ($\:{\lambda\:}_{i}=0.319{-}0.377\text{ eV}$), accelerating Marcus interfacial electron injection kinetics. Furthermore, dyes **XI** and **XII** demonstrate exothermic bidentate bridging chemisorption ( $\:\varDelta\:{E}_{\mathrm{ads}}=-15.21\text{ to }-16.71\text{ kcal/mol}$) with strong orbital coupling across the semiconductor interface. These findings establish a predictive computational framework for high-efficiency porphyrin photosensitizers.

**Supplementary Information:**

The online version contains supplementary material available at 10.1038/s41598-026-66026-x.

## Introduction

The global rate of energy consumption continues to rise rapidly, underscoring the urgent need for renewable and sustainable energy sources. Solar energy, as an abundant and eco-friendly resource, has emerged as a critical solution, with dye-sensitized solar cells (DSSCs) representing a prominent photovoltaic technology for converting light directly into electricity^1,2^. Among the core components of a DSSC, the photosensitizer plays a decisive role in dictating light absorption, interfacial charge separation, and overall device power conversion efficiency (PCE)^1^. Porphyrins and their transition metal complexes have gained immense prominence due to their exceptional light-harvesting capabilities across the visible and near-infrared (NIR) spectral regions, high thermal and chemical stability, facile structural tunability, and accessible synthetic pathways^2,3^. Recent molecular engineering breakthroughs demonstrate their remarkable potential, with engineered zinc-porphyrin dyes achieving power conversion efficiencies exceeding 13% when paired with cobalt-based redox electrolytes, directly rivaling conventional silicon-based photovoltaic architectures^3,4^.

The functional architecture of a standard DSSC comprises a transparent conducting oxide (TCO) photoanode coated with a mesoporous nanocrystalline semiconductor film (typically TiO_2_), an adsorbed monolayer of sensitizer molecules, an electrolyte solution containing a regenerative redox couple (such as $\:{\mathrm{I}}^{-}/{\mathrm{I}}_{3}^{-}$or cobalt/copper complexes), and a catalytic counter electrode^1,5,6^. Sensitizers broadly fall into two main categories: metal-based coordination complexes (e.g., ruthenium polypyridyl and zinc-porphyrin architectures) and metal-free organic dyes^6–8^. Metal-free organic dyes have attracted significant interest due to their high molar extinction coefficients, structural versatility, and low production costs, whereas macrocyclic metal complexes like zinc-porphyrins offer superior macrocyclic rigidity, broad spectral absorption, and exceptional long-term stability^6–8^. Optimizing device performance relies heavily on tuning intramolecular charge transfer (ICT) through push–pull (D–π–A) molecular architectures, where an electron-donating group (D) drives photoinduced electron density through a conjugated π-spacer (π) to an electron-withdrawing anchoring group (A) chemisorbed on the semiconductor surface^6,9–12^. This structural design fundamentally governs the short-circuit current density (J_sc_), open-circuit voltage (V_oc_), and charge injection efficiency ($\:{\varPhi\:}_{\mathrm{inj}})$ of the cell.

The optoelectronic and photophysical properties of porphyrin dyes can be systematically tailored through strategic peripheral substitution at the *meso* or $\:\beta\:$ positions of the macrocyclic ring^2,3,13–16^. Introducing electron-donating groups, such as amino (-NH_2_) or alkylamine moieties at the *para* position of terminal phenyl rings, destabilizes the highest occupied molecular orbital (HOMO), narrows the fundamental HOMO–LUMO energy gap (E_g_), red-shifts the optical absorption spectrum, and optimizes excited-state electron transfer to the TiO_2_ photoanode^13–15^. Conversely, introducing electron-withdrawing carboxylic acid (-COOH) or cyanoacrylic acid anchoring groups facilitates strong, spontaneous chemisorption onto the TiO_2_ surface through bidentate or bridging modes, ensuring strong spatial electronic coupling between the dye’s lowest unoccupied molecular orbital (LUMO) and the titanium 3d-conduction band while suppressing dark-current charge recombination^13–15^. Prior experimental and theoretical studies demonstrate that such structural design principles allow simplified zinc-porphyrin sensitizers to achieve PCEs in the range of 5.3%–7.1% with scalable synthetic routes^13–15^. Furthermore, recent advancements in asymmetric donor–acceptor configurations and dual-anchoring frameworks have proven highly effective in modulating charge transfer kinetics and preventing detrimental dye aggregation^11,12,17–20^. Numerous theoretical studies on porphyrin dye sensitizers have explored their electronic structures, working principles, and photovoltaic parameters^16,21–26^.

**Fig. 1:**
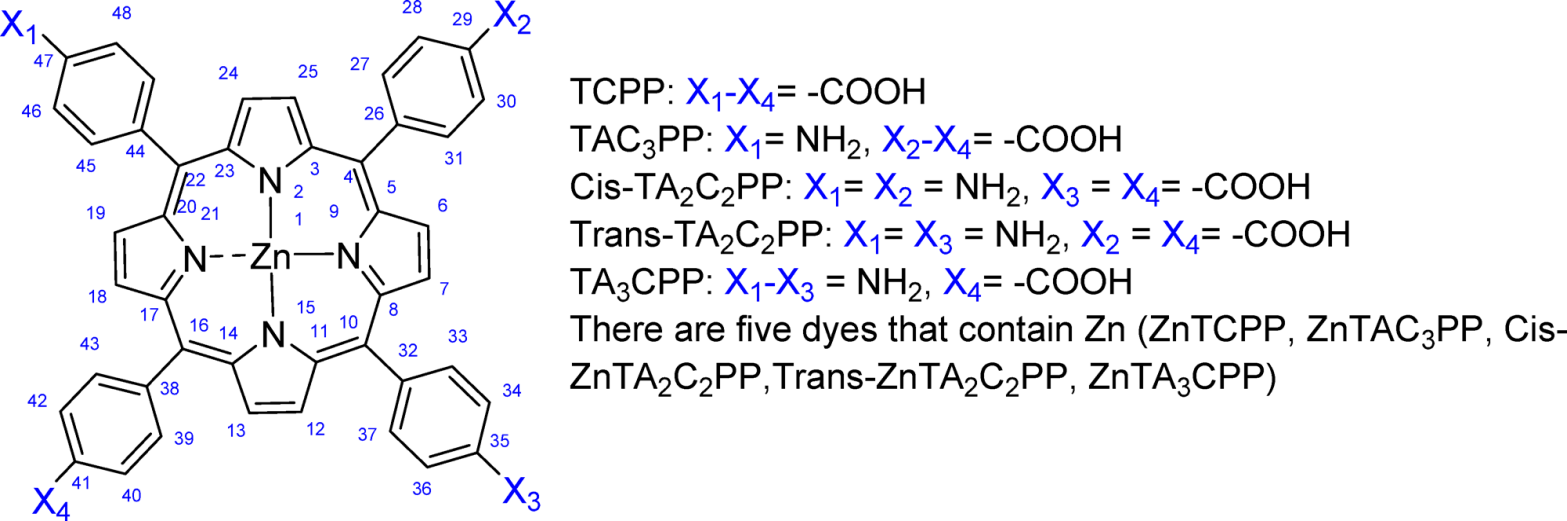
Chemical structures of the porphyrin ligands and the corresponding Zn complexes under study. The free ligands are abbreviated from dye **I** to dye **V**. The zinc-porphyrin dyes are abbreviated from dye **VI** to dye **X** in this sequence.

Metalloporphyrin dyes exhibit two distinct electronic absorption bands that govern their light-harvesting profile in DSSCs: an intense Soret (B) band in the near-UV/blue region (250–500 nm) and a weaker series of Q-bands in the visible/red region (550–700 nm), as described by Gouterman’s classical four-orbital model^27^. Enhancing photon-to-current conversion requires broadening and donor-intensifying the inherently weak Q-band absorption to achieve panchromatic visible light harvesting. Quantum chemical modeling using density functional theory (DFT) and time-dependent DFT (TD-DFT) provides invaluable atomistic insights into the excited-state electronic transitions, charge transfer metrics, and interfacial energetics that dictate solar cell performance^28,29^. In this study, a comparative quantum chemical evaluation is performed on a systematic series of ten baseline free-base (**I–V**) and zinc-complexed (**VI–X**) tetraphenylporphyrin (TPP) dyes carrying varying ratios and geometric configurations (*cis* vs. *trans*) of amino (-NH_2_) donor and carboxylate (-COOH) acceptor groups (Fig. 1)^13,30,31^. Gas-phase and solvated electronic structures, frontier molecular orbital distributions, and vertical excitation profiles were evaluated using hybrid B3LYP and long-range corrected CAM-B3LYP functionals combined with the LANL2DZ basis set^28,29^, which incorporates effective core potentials (ECPs) for the central zinc ion while providing a balanced description of the organic macrocycle environment. Bulk environmental dielectric influences were modeled using the Conductor-like Polarizable Continuum Model (C-PCM) across THF and EtOH media^32^.

This study aims to explore the direct relationships between chemical structure, peripheral substitution topology, and molecular electronic properties across these sensitizers.

**Fig. 2:**
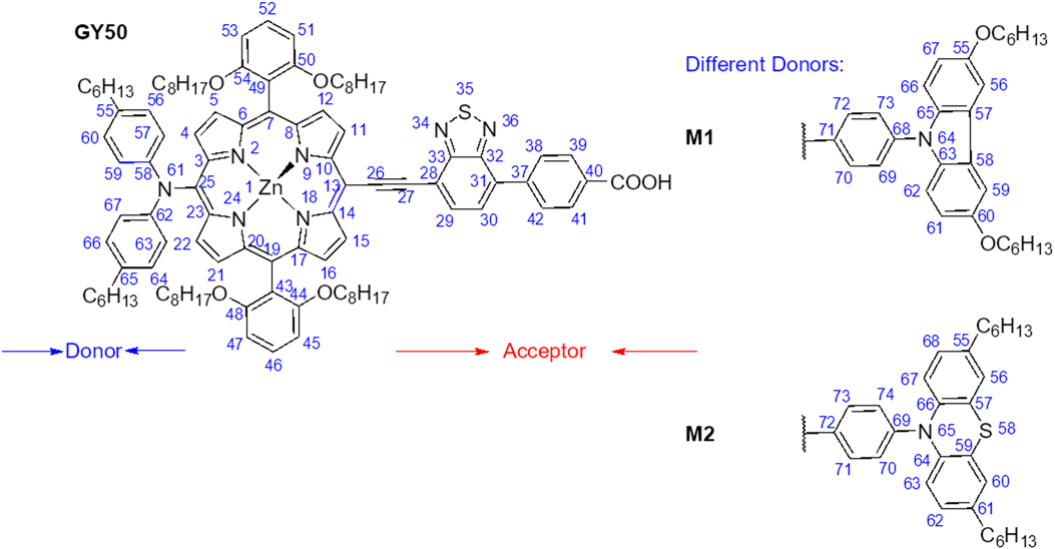
Chemical structures of the two newly modeled porphyrin dyes (**XI**,** XII**), sharing chemical similarity with modification of the donor moiety of the benchmark **GY50** dye.

Additionally, the impact of donor group modifications on the benchmark **GY50** sensitizer framework (depicted in Fig. 2) was explored^4,19,20,33^. Two novel asymmetric zinc-porphyrin dyes (**XI** and **XII**) were rationally designed by modifying the donor moiety and breaking macrocyclic symmetry $\:{(D}_{4\mathrm{h}}\to\:{C}_{1}$). Systematic donor substitution was analyzed to determine its effects on key parameters such as molar absorptivity, Q-band light harvesting, intramolecular charge transfer efficiency$\:{\:(q}_{CT},\:{d}_{CT}$), Marcus interfacial electron injection kinetics (k_injection_), and surface adsorption energetics on periodic anatase (TiO_2_)_36_(101) clusters^12,19,20,34–37^. Comparative evaluation of these modified target dyes concerning electronic properties and photovoltaic descriptors offers key insights into the design principles governing peripheral donor/acceptor topology, thereby guiding the computational design of high-efficiency porphyrin-based sensitizers for DSSC applications.

## Computational methodology

Quantum chemical calculations for isolated dye molecules (**I–XII**) were performed using the Gaussian 09 software suite^38^, following established DSSC computational protocols^12,19,20^. Ground-state geometries were fully optimized at the B3LYP/LANL2DZ level of theory. Single-point vertical excitation and excited-state photophysical properties were evaluated using time-dependent DFT (TD-DFT) with the long-range corrected CAM-B3LYP functional^28,29^. CAM-B3LYP incorporates long-range Hartree–Fock exchange $\:(\alpha\:+\beta\:=0.65\:)$to eliminate self-interaction errors and prevent the artificial underestimation of charge-transfer excitation energies in extended π-conjugated macrocycles^28,29^. The LANL2DZ basis set implements relativistic Effective Core Potentials (ECPs) for the central transition metal ion (Zn^2+^) while providing a balanced split-valence treatment for lighter atoms (C, H, N, O, S)^13,30,31^.

Solvent effects in EtOH and THF were explicitly modeled using the Conductor-like Polarizable Continuum Model (C-PCM) within the Self-Consistent Reaction Field (SCRF) framework^32^.

Quantitative wavefunctions and spatial intramolecular charge-transfer (ICT) parameters were analyzed using Multiwfn (version 3.8)^39,40^ based on the density-difference method proposed by Le Bahers, Adamo, and Ciofini^40^. The net transferred electronic charge ($\:{q}_{\mathrm{CT}}$) was computed by integrating the spatial electron density difference resulting from vertical photoexcitation ($\:\varDelta\:\rho\:\left(r\right)$) over positive ($\:{\rho\:}^{+}\left(r\right))\:$and negative ($\:{\rho\:}^{-}\left(r\right)$) regions:

1 $$\:\varDelta\:\rho\:\left(r\right)={\rho\:}_{\mathrm{EX}}\left(r\right)-{\rho\:}_{\mathrm{GS}}\left(r\right)$$

2 $$\:{q}_{\mathrm{CT}}=\int\:{\rho\:}^{+}\left(r\right)\hspace{0.17em}dr=\int\:|{\rho\:}^{-}\left(r\right)|\hspace{0.17em}dr$$

The charge-transfer distance ($\:{d}_{\mathrm{CT}}$) was evaluated as the Euclidean distance between the barycenters of the positive ($\:{r}^{+}$) and negative ($\:{r}^{1}$) density distributions^39,40^:

3 $$\:{d}_{\mathrm{CT}}=\left|{r}^{+}-{r}^{-}\right|$$

### Quantum chemical descriptors

Conceptual DFT reactivity descriptors—global chemical potential (µ), global chemical hardness (η), ionization energy (I), electron affinity (A), electronegativity ($\:{\upchi\:}$) and electrophilicity index (ω)—alongside light-harvesting efficiency (LHE) were calculated directly from boundary molecular orbital energies^41,42^:

4 $$\:\mu\:={\left(\frac{\partial\:E}{\partial\:N}\right)}_{v\left(r\right)}\approx\:-\:\frac{I+A}{2}=\:\frac{-{E}_{HOMO}+(-{E}_{LUMO}\:)}{2}\:=\:-\:{\upchi\:}$$

5 $$\:\eta\:={\left(\frac{\partial\:\mu\:}{\partial\:N}\right)}_{v\left(r\right)}\approx\:\frac{I-A}{2}$$

6 $$\:\omega\:=\frac{{\mu\:}^{2}}{2\eta\:}$$

7 $$\:\mathrm{L}\mathrm{H}\mathrm{E}\:{(}_{\mathrm{a}\mathrm{t}}{{\uplambda\:}}_{\mathrm{m}\mathrm{a}\mathrm{x}.})\:=1-{10}^{-\:\mathrm{o}\mathrm{s}\mathrm{c}\mathrm{i}\mathrm{l}\mathrm{l}\mathrm{a}\mathrm{t}\mathrm{o}\mathrm{r}\:\mathrm{s}\mathrm{t}\mathrm{r}\mathrm{e}\mathrm{n}\mathrm{g}\mathrm{t}\mathrm{h}\:\left(f\right)}$$

Where E_HOMO_ is the highest occupied molecular orbital energy, E_LUMO_ is the lowest unoccupied molecular orbital energy, * f* is the dimensionless electronic oscillator strength at the maximum absorption wavelength (λ_max_.)^41,42^.

### Photoelectrochemical parameters and conduction band shifts

The maximum achievable open-circuit voltage (V_OC_) and semiconductor conduction band edge shifts ($\:\varDelta\:{E}_{\mathrm{CB}}$) were evaluated as^43^:

8 $$\:{V}_{OC}=\frac{{E}_{CB}+{\varDelta\:E}_{CB}}{e}-{E}_{redox}+\frac{{K}_{B}T}{e}\left(ln\frac{{J}_{SC}}{{J}_{o}}\right)$$

9 $$\:\varDelta\:{E}_{\mathrm{CB}}=\frac{e\cdot\:{\mu\:}_{n}\cdot\:\gamma\:}{{\epsilon\:}_{r}\cdot\:{\epsilon\:}_{0}}$$

Where $\:{E}_{CB}$is the intrinsic conduction band edge position of TiO_2_ (taken as -4.0 eV versus vacuum), e is the elementary charge, $\:{K}_{B}$ is the Boltzmann constant, T is the absolute temperature (298.15 K), $\:{J}_{SC}$ is the operating short-circuit current density, $\:{J}_{o}$ signifies the reverse saturation dark current density, $\:{\mu\:}_{n}$ is the component of the dipole moment of the adsorbed dye perpendicular to the semiconductor surface, $\:\gamma\:$ is the surface coverage, $\:{\epsilon\:}_{0}$ is the vacuum permittivity, and $\:{\epsilon\:}_{r}$is the effective dielectric constant of the organic monolayer^43^.

### Thermodynamic driving forces

Thermodynamic driving forces for electron injection ($\:{\varDelta\:G}_{injection}$) and dye regeneration ($\:{\varDelta\:G}_{regeneration})$ were modeled according to according to Rehm–Weller electrochemical protocols^44^:

10 $$\:{\varDelta\:G}_{injection}={E}_{OX}^{{dye}^{*}}-{E}_{CB}\:where\:\:\:\:\:{E}_{OX}^{{dye}^{*}}={E}_{OX}^{dye}-{E}_{0-0}^{dye}$$

11 $$\:\:\:\:\:\:\:\:\:\:\:\:\:{\varDelta\:G}_{regeneration}={E}_{OX}^{dye}-{E}_{redox\:\left(electrolyte\right)}={E}_{OX}^{dye}-(-4.85\:\:eV)$$

Where $\:{E}_{OX}^{dye}$ is the ground-state oxidation potential (assumed equal to -E_HOMO_), $\:{E}_{OX}^{{dye}^{*}}$is the excited-state oxidation potential, and $\:{E}_{0-0}^{dye}\:\:\:$ represents the vertical electronic transition energy corresponding to the absorption maximum ($\:{\lambda\:}_{max}$). The redox potential of the standard $\:{\mathrm{I}}^{-}/{\mathrm{I}}_{3}^{-}\:$electrolyte system( $\:{E}_{redox\:\left(electrolyte\right)}$) is taken as -4.85 eV relative to vacuum^44^.

### Interfacial electron transfer kinetics

The interfacial electron injection rate constant ($\:{k}_{injection}$) was modeled via non-adiabatic Marcus electron-transfer theory^45–48^:

12 $$\:{k}_{injection}=A{e}^{\left[-\frac{{\left({\varDelta\:G}_{injection}+\lambda\:\right)}^{2}}{4\lambda\:{K}_{B}T}\right]}$$

Where A is a pre-factor that depends on the strength of the electronic dye-surface coupling ($\:A=\frac{2\pi\:}{\hslash\:}{\left|{H}_{DA}\right|}^{2}\rho\:\left({E}_{CB}\right)\frac{1}{\sqrt{4\pi\:\lambda\:{K}_{B}T}}$, K_B_ is the Boltzmann constant, T is the absolute temperature, and λ is the total reorganization energy:

13 $$\:{\uplambda\:}={\lambda\:}_{i}+{\lambda\:}_{o}$$

Where $\:{\lambda\:}_{i}$ is the internal molecular reorganization energy of the dye in the initial electronic ground state (g.s) evaluated at the relaxed structures corresponding to initial and final electronic states in vacuum, and $\:{\lambda\:}_{o}\:$is the outer-sphere reorganization energy associated with the solvent structural response to the change in electronic charge on the solvated dye molecules. J_sc_ is enhanced by improving LHE and $\:{\varPhi\:}_{inj}$, as well as by decreasing the total reorganization energy.

14 $$\:{\lambda\:}_{i}={\lambda\:}_{h}+{\lambda\:}_{e}=\left[{E}_{\mathrm{ion}}\left({Q}_{0}\right)-{E}_{\mathrm{ion}}\left({Q}_{\mathrm{ion}}\right)\right]+\left[{E}_{0}\left({Q}_{\mathrm{ion}}\right)-{E}_{0}\left({Q}_{0}\right)\right]$$

where $\:{\lambda\:}_{h}$ and $\:{\lambda\:}_{e}$ are hole and electron reorganization energies, $\:{E}_{\mathrm{ion}}$ is the total energy of the charged state, and $\:{E}_{\mathrm{0}}$ is the energy of the neutral molecule evaluated at neutral ($\:{Q}_{0}$) and relaxed ionic ($\:{Q}_{\mathrm{ion}}$) geometries^45^.

### Solid-state interfacial modeling and adsorption energetics

Surface chemisorption onto the dominant anatase TiO_2_(101) facet was investigated using a stoichiometric (TiO_2_)_36_ cluster model within the DMol³ package in Materials Studio^34–37^. Surface calculations utilized the Generalized Gradient Approximation (GGA) with the Perdew–Burke–Ernzerhof (PBE) functional and numerical Double Numerical plus d-functions (DNP) basis sets^34,37^. The (TiO_2_)_36_ cluster provides a 1.5 nm x 1.5 nm active area, allowing stable bidentate bridging chemisorption without edge-truncation artifacts^35,36,49^.

The adsorption energy ($\:{\varDelta\:E}_{ads}$) was evaluated as^35–37^:

15 $$\:{\varDelta\:E}_{ads}={E}_{dye+Ti{O}_{2}}-{E}_{dye}-{E}_{Ti{O}_{2}}$$

where $\:{E}_{dye+Ti{O}_{2}}\:$is the total energy of the adsorbed complex, $\:{E}_{dye}$is the energy of the isolated dye, and $\:{E}_{Ti{O}_{2}}$= -4,719,462.79 kcal/mol is the total energy of the clean, relaxed (TiO_2_)_36_ cluster. A negative $\:{\varDelta\:E}_{ads}\:$indicates spontaneous, exothermic chemisorption^35–37^.

## Results and discussion

The ground-state properties and quantum chemical parameters of all investigated dyes (**I–XII**) are systematically evaluated using Roman numerals across all discussions and tables. Dyes **I–V** represent the free-base porphyrins, while dyes **VI–X** denote their corresponding zinc-metalated complexes, originally synthesized and spectroscopically characterized by Walter et al. and Rudine et al.^13,30,31^. Building upon these experimental frameworks, dyes **XI** and **XII** represent our newly designed, asymmetric zinc-porphyrin models featuring highly conjugated, electron-donating amine moieties.

### Ground-state geometrical analyses of dyes I–XII

The optimized ground-state geometries of the free-base (**I–V**) and zinc-complexed (**VI–XII**) porphyrin dyes are summarized in Table 1. Across all zinc complexes (**VI–XII**), the central zinc–porphyrin macrocycle exhibits a highly planar geometry, with internal N_21_–Zn–N_15_ bond angles confined to a narrow range ($\:{89.88}^{\circ\:}{-}{90.11}^{\circ\:}$). This rigid coordination environment preserves macrocyclic structural integrity and facilitates extensive π- electron delocalization to promote intramolecular charge transport^13,31^.

In contrast, the peripheral *meso*-phenyl substituents are substantially rotated out of the macrocyclic plane, displaying average C_17_–C_16_–C_38_–C_39_ dihedral angles of $\:{63.63}^{\circ\:}{-}{68.98}^{\circ\:}$(and $\:{114.95}^{\circ\:}{-}{116.04}^{\circ\:}$ for specific *meso* orientations). This non-coplanar arrangement introduces significant steric hindrance that physically suppresses face-to-face ($\:\pi{-}\pi\:)$molecular stacking on the semiconductor photoanode surface, thereby minimizing excited-state self-quenching and dye aggregation^13,30^.

**Table 1:** Selected ground-state optimized geometrical parameters for dyes **I–XII** calculated using B3LYP/ LANL2DZ level of theory in gas phase.

Dye	TCPP (I) (ZnTCPP) (VI)	TAC_3_PP (II) (ZnTAC_3_PP) (VII)	Cis-TA_2_C_2_PP (III) (Cis- ZnTA_2_C_2_PP) (VIII)	Trans-TA_2_C_2_PP (IV) (Trans-ZnTA_2_C_2_PP) (IX)	TA_3_CPP (V) (ZnTA_3_CPP) (X)	M1 (XI)	M2 (XII)
Bond: Distances Å							
Zn-N_21_	(2.07)	(2.07)	(2.07)	(2.07)	(2.07)	2.07	2.07
(C = O)^a^	1.24 (1.24)	1.24 (1.24)	1.25 (1.25)	1.25 (1.25)	1.25 (1.25)	1.25	1.25
(C-O)^a^	1.39 (1.39)	1.39 (1.39)	1.39 (1.39)	1.39 (1.39)	1.39 (1.39)	1.39	1.39
(C_47_-N)^b^	-	1.39 (1.39)	1.39 (1.39)	1.39 (1.39)	1.40 (1.40)	1.43	1.45
Angles °							
N_21_-Zn-N_15_	(90.02)	(90.08)	(90.00)	(90.11)	(90.09)	89.88	89.92
(O = C-O)^a^	121.26 (121.24)	121.16 (121.13)	121.05 (121.04)	121.08 (121.07)	120.95 (120.94)	120.93	120.95
(H-N-H)^b^	-	118.13 (118.14)	118.18 (118.19)	118.15 (118.16)	118.20 (118.21)	-	-
Dihedral Angles°							
C_17_-C_16_-C_38_ -C_39_	-64.07 (-64.08)	115.67 (116.04)	115.20 (114.95)	-63.67 (-63.63)	68.11 (68.98)	65.52	68.15
C_8_-C_10_-C_32_-C_33_	-115.80 (-115.91)	64.43 (64.28)	110.95 (110.58)	-116.54 (-116.24)	64.00 (63.98)	65.52	68.15

a Carboxylic acid anchoring group parameter.

b Amino donor group parameters.

(Values in parentheses correspond to the respective metal-free ligands)

### Quantum chemical and reactivity indices

Table 2 details the calculated dipole moments ($\mu$), frontier molecular orbital energies ($$\:{E}_{\mathrm{HOMO}}$$,$$\:{E}_{\mathrm{LUMO}}$$, fundamental HOMO–LUMO energy gaps (E_g_), and net solvation stabilization energies $$\:\varDelta\:{E}_{\mathrm{solvation}}={E}_{\mathrm{solvated}}-{E}_{\mathrm{gas}}$$) obtained under the range-separated CAM-B3LYP/LANL2DZ framework with C-PCM solvation^29,32^. To streamline data presentation and avoid redundancy, the corresponding comparative benchmark dataset computed at the B3LYP/LANL2DZ level has been arranged separately in Table S1 of the Supporting Information. The calculated exothermic $$\:\varDelta\:{E}_{\mathrm{solvation}}$$ values are consistently larger in polar EtOH $$\:(-22.00\text{ to }-31.63\text{ kcal/mol}$$) than in THF ($$\:-19.56\text{ to }-28.27\text{ kcal/mol}$$), indicating that the polar, protic environment of ethanol provides superior electrostatic stabilization for these mixed-substituent macrocycles^32^.

Across the baseline series (**I–X**), the fundamental HOMO–LUMO gap (E_g_) stabilizes between 4.12 eV and 4.45 eV in solution. In contrast, target dyes **XI** and **XII** display significantly narrowed HOMO–LUMO gaps of 3.68 eV and 3.70 eV in THF, respectively. This HOMO–LUMO gap narrowing is driven by the introduction of highly conjugated, electron-rich donor moieties at the peripheral *meso*-position, which destabilizes the ground-state HOMO level relative to the baseline frameworks^13,31^.

The electronic energy level alignments relative to the device architecture reveal that the $\:{E}_{\mathrm{LUMO}}\:$ levels for all twelve sensitizers $\:(-1.44\text{ to }-2.47\text{ eV}$) are positioned well above the intrinsic conduction band edge of the anatase TiO_2_(101) photoanode $\:{(\:E}_{\mathrm{CB}}=-4.00\text{ eV}$versus vacuum)^43,44^. This energetic positioning establishes a thermodynamically favorable driving force for downhill excited-state electron injection into the semiconductor $\:(\varDelta\:{G}_{\mathrm{injection}}<0$). Concurrently, the ground-state $\:{E}_{\mathrm{HOMO}}\:$levels $\:-5.71\text{ to }-6.58\text{ eV}$) lie deeply below the Nernstian redox potential of the standard $\:{\mathrm{I}}^{-}/{\mathrm{I}}_{3}^{-}$ electrolyte couple $\:({E}_{\mathrm{redox}}=-4.85\text{ eV}\mathrm{),}$ensuring rapid ground-state dye regeneration $\:(\varDelta\:{G}_{\mathrm{regeneration}}<0$) and suppressing dark-current charge recombination^44^.

Conceptual DFT reactivity descriptors further illuminate the charge-separation tendencies^41,42^. Dyes **V**,** X**, and the target models **XI** and **XII** yield the lowest ω in the series, confirming their strong photosensitization potential. Furthermore, trans-configured dyes **IV** and **IX**, alongside target models **XI** and **XII**, exhibit low global chemical hardness $\:(\eta\:=1.73\mathrm{--}2.51\text{ eV}$), indicating a highly polarizable electronic cloud that facilitates intramolecular charge separation upon photoexcitation^41,42^. Calculated µ follow the decreasing sequence $\:\mathbf{X}\mathbf{I}\mathbf{I}>\mathbf{X}\mathbf{I}>\mathbf{V}>\mathbf{X}\:$, reflecting an enhanced electron-escaping tendency from the ground state that favors electron injection kinetics.

**Table 2:** Computed energetic parameters, µ, HOMO–LUMO gaps (E_g_) and solvation stabilization energies $\:\left(\varDelta\:{E}_{\mathrm{solvation}}\:\right)$for Dyes **I–XII** calculated at the CAM-B3LYP/LANL2DZ level with C-PCM solvation.

Dye	Medium	E_Total_ (au)	µ (D)	E_HOMO_ (eV)	E_LUMO_ (eV)	HOMO-LUMO gap (eV)	∆E _solvation_ (kcal/mol)
**I**	GAS	-2666.27	1.66	-6.52	-2.17	4.35	-
	THF	-2666.30	2.10	-6.57	-2.22	4.35	-21.53
	ETOH	-2666.31	2.18	-6.58	-2.23	4.35	-24.11
**II**	GAS	-2533.12	4.93	-6.22	-1.97	4.25	-
	THF	-2533.15	5.75	-6.36	-2.14	4.22	-20.65
	ETOH	-2533.15	5.29	-6.38	-2.17	4.21	-23.15
**III**	GAS	-2399.93	8.98	-5.95	-1.77	4.18	-
	THF	-2399.99	10.02	-6.21	-2.02	4.19	-20.59
	ETOH	-2399.96	10.29	-6.24	-2.12	4.12	-22.55
**IV**	GAS	-2399.95	4.29	-6.07	-1.87	4.20	-
	THF	-2399.98	4.65	-6.25	-2.09	4.16	-23.17
	ETOH	-2399.99	4.78	-6.27	-2.12	4.15	-26.05
**V**	GAS	-2266.81	5.97	-5.71	-1.51	4.20	-
	THF	-2266.84	6.94	-6.08	-1.94	4.14	-19.56
	ETOH	-2266.84	6.75	-6.13	-2.00	4.13	-22.00
**VI**	GAS	-2730.65	1.69	-6.55	-2.10	4.45	-
	THF	-2730.70	2.12	-6.46	-2.04	4.42	-26.49
	ETOH	-2730.70	2.19	-6.44	-2.03	4.41	-29.59
**VII**	GAS	-2597.50	4.66	-6.24	-1.91	4.33	-
	THF	-2597.54	5.14	-6.26	-1.97	4.29	-25.84
	ETOH	-2597.55	5.18	-6.26	-1.98	4.28	-28.85
**VIII**	GAS	-2464.34	8.49	-6.01	-1.64	4.37	-
	THF	-2464.38	9.64	-6.13	-1.84	4.29	-24.79
	ETOH	-2464.39	9.77	-6.14	-1.86	4.28	-27.69
**IX**	GAS	-2464.33	4.34	-6.09	-1.80	4.29	-
	THF	-2464.38	4.68	-6.16	-1.92	4.24	-28.27
	ETOH	-2464.38	4.71	-6.17	-1.93	4.24	-31.63
**X**	GAS	-2331.19	5.84	-5.74	-1.44	4.30	-
	THF	-2331.23	6.60	-5.99	-1.76	4.23	-24.53
	ETOH	-2331.23	6.69	-6.03	-1.80	4.23	-27.42
**XI**	GAS	-5285.90	11.47	-5.79	-2.25	3.54	-
	THF	-5285.96	4.70	-6.14	-2.46	3.68	-35.76
**XII**	GAS	-5145.59	11.45	-5.82	-2.26	3.56	-
	THF	-5145.64	5.42	-6.17	-2.47	3.70	-32.94

### Frontier molecular orbitals and intramolecular charge-transfer characterization

**Fig. 3:**
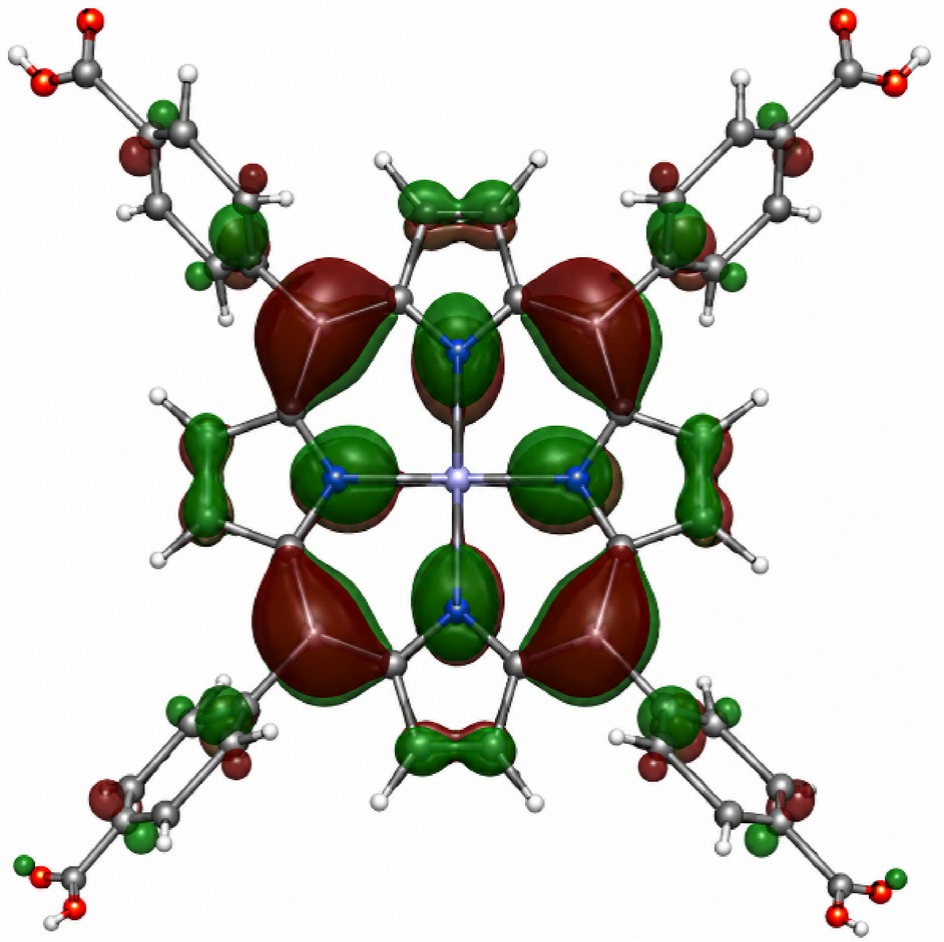
Representative 3D frontier molecular orbital spatial electron density distributions for Dyes **I–X** calculated in ethanol at an isovalue of 0.02 au (CAM-B3LYP/LANL2DZ/C-PCM). Green and red lobes denote positive and negative phases of the molecular wavefunctions, respectively. *(Detailed wavefunction plots for all additional states are provided in Fig.**S1** of supplementary information (SI)*.

To elucidate photoinduced electronic transition mechanisms, 3D frontier molecular orbital (FMO) spatial electron density distributions were evaluated at an isovalue of 0.02 au in EtOH and visualized in Fig. 3^28,29,39^.

The spatial topology of these boundary orbitals provides critical insight into ICT characteristics within the $\:\mathrm{D-}\pi\:{\text {-A}}\text{}$architecture across dyes **I–X**. The HOMO electron density is localized predominantly across the conjugated porphyrin core and the peripheral, electron-rich -NH_2_ donor groups, where nitrogen lone pairs actively participate in extending π-electron delocalization to elevate the ground-state energy level^13,30^. Upon vertical photoexcitation, a pronounced spatial charge migration occurs electron density in the LUMO and LUMO + 1 states shifts completely away from the donor moieties and macrocyclic core, concentrating overwhelmingly onto the terminal -COOH anchoring groups. This clear spatial segregation between HOMO and LUMO states confirms a strong, directional ICT process^40^. Furthermore, selective localization of excited-state wavefunctions directly on the anchoring carboxylates ensures substantial electronic overlap with titanium 3d-orbitals on the semiconductor surface, facilitating ultrafast interfacial electron injection into the TiO_2_ photoanode^5,46^.

**Fig. 4:**
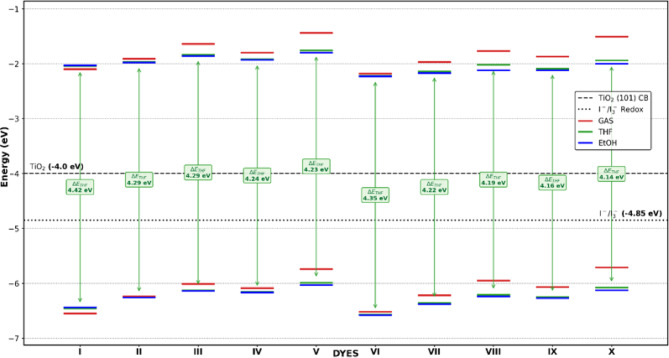
Schematic energy band diagram illustrating the calculated HOMO and LUMO energy levels of the studied dyes in the gas phase and THF solvent at the CAM-B3LYP/LANL2DZ level relative to the anatase TiO_2_ conduction band edge and the $\:{\mathrm{I}}^{-}/{\mathrm{I}}_{3}^{-}$ electrolyte redox potential. Note: The fundamental HOMO–LUMO energy gaps (E_g_) indicated by the green lines correspond specifically to the values calculated in the THF solvent medium.

Operational DSSC efficacy is fundamentally dictated by the relative alignment of dye FMO levels with the conduction band edge of the TiO_2_(101) photoanode $\:{E}_{\mathrm{CB}}=-4.00\text{ eV}$ versus vacuum) and the Nernstian redox potential of the electrolyte $\:{E}_{\mathrm{redox}}=-4.85\text{ eV}$)^43,44^. Figure 4 illustrates the schematic energy level alignment of $\:{E}_{\mathrm{HOMO}}$and $\:{E}_{\mathrm{LUMO}}\:$for dyes **I–X** across gas, THF, and EtOH phases. Across all media, $\:{E}_{\mathrm{LUMO}}\:$levels for both free-base (**I–V**) and zinc-metalated (**VI–X**) porphyrins are positioned well above $\:{E}_{\mathrm{CB}}$, guaranteeing exothermic excited-state electron injection ($\:\varDelta\:{G}_{\mathrm{injection}}<0$). As the ratio of -NH_2_ donors to -COOH anchors increases (moving from dye **I** to **V** and **VI** to **X**), $\:{E}_{\mathrm{LUMO}}$ levels shift upward toward vacuum (moving from − 2.17 eV in dye **I** to -1.44 eV in dye **V** in the gas phase), enhancing the thermodynamic driving force for electron injection into the semiconductor^44^.

Concurrently, ground-state $\:{E}_{\mathrm{HOMO}}$ levels for all ten sensitizers $\:-5.71\text{ to }-6.58\text{ eV}$) lie deeply below $\:{E}_{\mathrm{redox}}$ establishing a robust energetic driving force for dye regeneration $\:(\varDelta\:{G}_{\mathrm{regeneration}}<0$) by $\:{\mathrm{I}}^{-}\:$and preventing dark-current charge recombination^43,44^. Solvent media induce electrostatic stabilization, pulling both $\:{E}_{\mathrm{HOMO}}$and $\:{E}_{\mathrm{LUMO}}$ levels downward relative to the gas phase, with polar EtOH providing slightly greater stabilization than THF^32^. Insertion of the central Zn^2+^ ion induces minor orbital stabilization while preserving the required energetic alignment for efficient operational cycles^13,31^.

### Optical properties and FMO analyses: target dyes (XI, XII) vs. GY50 benchmark

To quantitatively evaluate photoinduced charge separation, wavefunction decomposition analysis was performed on boundary orbitals. Table 3 details computed orbital energies, HOMO–LUMO gaps (E_g_), and fractional percentage contributions of donor and acceptor moieties for the benchmark sensitizer **GY50**^4,33^ alongside target zinc-porphyrins **XI** (Model 1) and **XII** (Model 2) at the CAM-B3LYP/LANL2DZ level.

**Table 3:** Calculated Orbital Energies, Energy Gaps (E_g_), and Fractional Molecular Orbital Contributions (%) of the Donor and Acceptor Moieties for Dyes **GY50**,** XI**, and **XII** (CAM-B3LYP/LANL2DZ).

Dye	Medium	E_HOMO_ (eV)	E_LUMO_ (eV)	Eg (eV)	Donor% HOMO	Acceptor% HOMO	Donor% LUMO	Acceptor% LUMO
GY50	GAS	-6.42	-2.15	4.27	20	80	1	99
	THF	-6.48	-2.20	4.28	24	76	1	99
XI	GAS	-6.14	-2.46	3.68	7	93	0	100
	THF	-6.14	-2.46	3.68	7	93	1	99
XII	GAS	-6.17	-2.47	3.70	4	96	0	100
	THF	6.17	-2.47	3.70	4	96	1	99

In THF solvent, the fractional donor contribution to the ground-state HOMO is 24% for **GY50**^33^ but drops to 7% for **XI** and 4% for **XII**. This reduction confirms that ground-state electron density in the target dyes is centralized within the macrocyclic core and auxiliary 2,1,3-benzothiadiazole (BTD) bridge. Concurrently, the acceptor segment contribution to the LUMO reaches 99% across all three sensitizers in solution, establishing that vertical excitation concentrates electronic density over the carboxylate anchor to facilitate interfacial injection^40^.

**Fig. 5:**
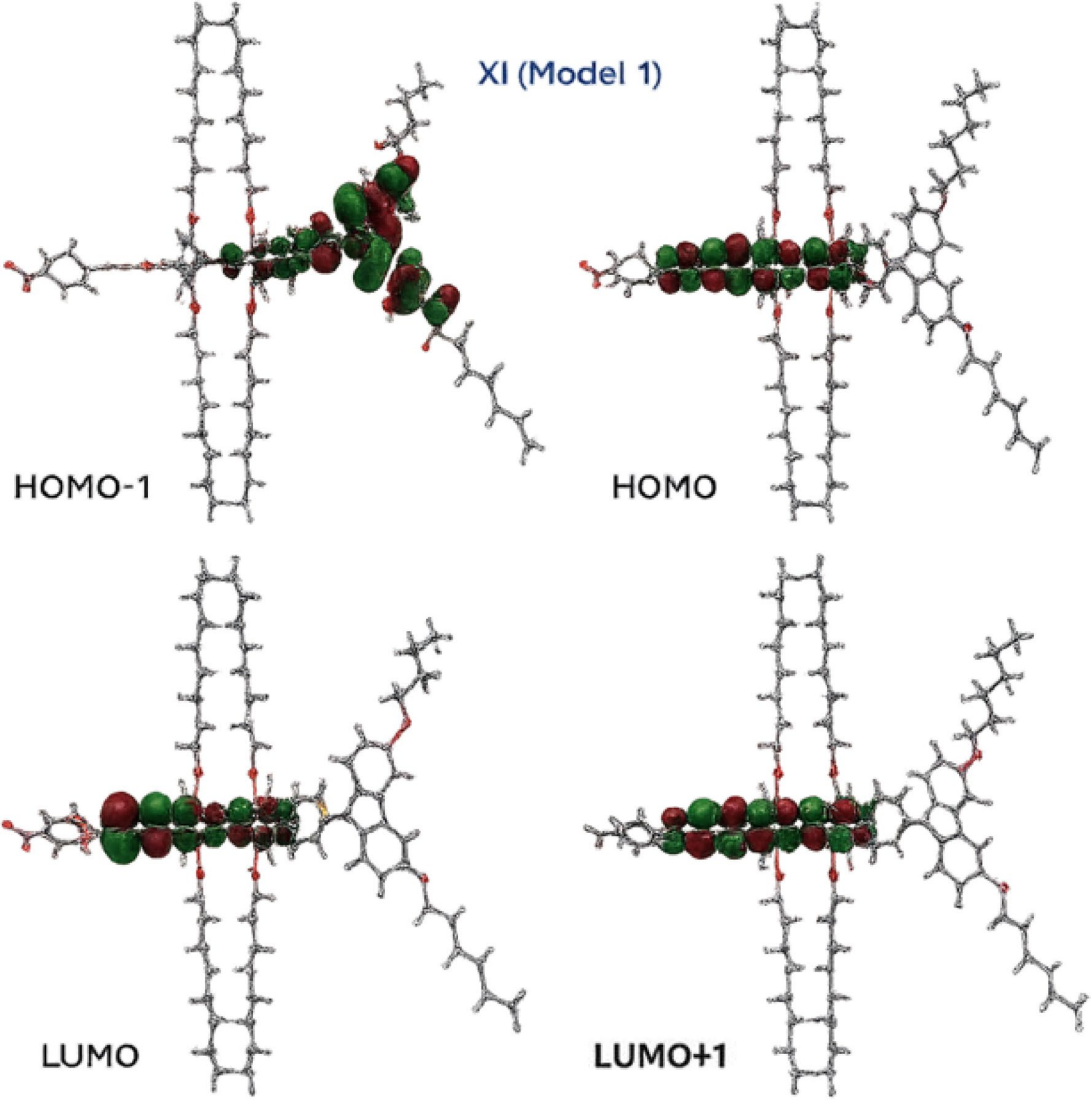
Spatial electronic density distributions (isovalue = 0.02 au) of Gouterman frontier molecular orbitals (HOMO-1, HOMO, LUMO, LUMO + 1) for target modeled Dye **XI** calculated at the CAM-B3LYP/LANL2DZ level of theory.

**Fig. 6:**
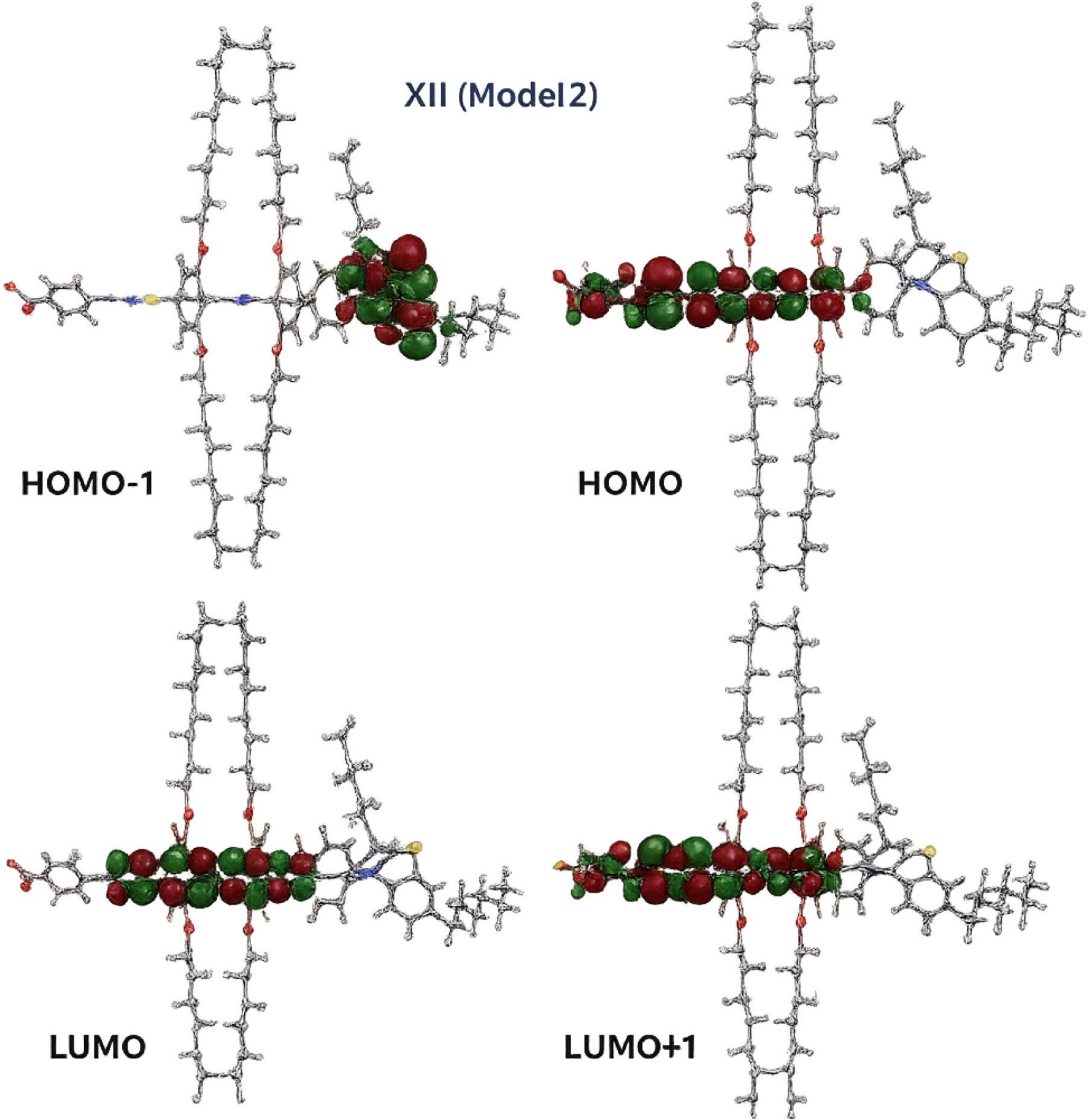
Spatial electronic density distributions (isovalue = 0.02 au) of Gouterman frontier molecular orbitals (HOMO-1, HOMO, LUMO, LUMO + 1) for target modeled Dye **XII** calculated at the CAM-B3LYP/LANL2DZ level of theory, illustrating spatial redistribution induced by the polarizable sulfur atom in the donor framework.

Spatial 3D electron density distributions (HOMO-1, HOMO, LUMO + 1) for target dyes **XI** and **XII** evaluated at an isovalue of 0.02 au are depicted in Figs. 5 and 6, respectively. For **GY50**, the HOMO-1 density is localized over the porphyrin macrocycle, whereas the HOMO density is delocalized across the donor, porphyrin core, and BTD auxiliary acceptor^4,33^. In target dye **XI** (Fig. 5), the HOMO-1 density is concentrated over the electron-donating unit with extension into the porphyrin ring, while the HOMO state is concentrated across the macrocycle and conjugated BTD bridge. In target dye **XII** (Fig. 6), incorporating a softer, more polarizable sulfur atom within the donor framework increases local electron density, driving a spatial redistribution of HOMO-1 density onto the central macrocycle in the HOMO state. For both target dyes **XI** and **XII**, unoccupied LUMO wavefunctions are localized over the porphyrin core and terminal acceptor/anchoring segment, confirming efficient directional ICT upon photoexcitation^40^.

Metalloporphyrin optical absorption is governed by Gouterman’s four-orbital model^27^. Configuration interaction among transitions involving boundary orbitals (HOMO-1, HOMO, LUMO, LUMO + 1) yields two low-energy Q-bands in the visible spectrum alongside one high-energy Soret (B) band in the near-UV/blue region^27^. Single-particle $\:\mathrm{HOMO}\to\:\mathrm{LUMO}$ transitions generate primary $\:{S}_{0}\to\:{S}_{1}\:$(Q-band) absorption, whereas higher-lying Q-states involve $\:\mathrm{HOMO}\to\:\mathrm{LUMO+1}\text{}$excitations. The high-intensity Soret (B) band ( $\:{S}_{0}\to\:{S}_{2}$) originates from deeper occupied levels (HOMO-1, HOMO-2) terminating at vacant LUMO or LUMO+2 states, as detailed by TD-DFT assignments in Table 4^28,29^.

**Table 4:** Calculated TD-DFT Optical Parameters, Maximum Absorption Wavelengths (λ_max_), Transition Oscillator Strengths (* f*), and Major Orbital Contributions for Dyes **I–XII** (CAM-B3LYP/LANL2DZ/C-PCM).

Dye	Medium	λ_max_ (nm)	ƒ	Contribution	Dye	λ_max_ (nm)	ƒ	Contribution
**I**	GAS	366.58(B) 606.54(Q)	1.92 0.03	H-L→L(30%) H→L(31.6%)	**VI**	369.30(B) 556.67(Q)	1.79 0.03	H-1→L + 1(28.8%) H→L (28.9%)
	THF	389.07(B) 604.97(Q)	2.20 0.06	H-1→L (30.9) H→L (32.6%)		392.13(B) 561.65(Q)	2.09 0.05	H-1→L + 1(30.1%) H→L (30.4%)
	ETOH	387.01(B) 604.17(Q)	2.19 0.06	H-1→L (30.7%) H→L (32.6%)		390.47(B) 561.79(Q)	2.06 0.05	H-1→L + 1(29.9%) H→L (30.4%)
**II**	GAS	371.31(B) 610.91(Q)	1.69 0.08	H-1→L + 1(19.1%) H→L (22.3%)	**VII**	376.42(B) 562.61(Q)	1.65 0.05	H-1→L (26%) H→L (30.3%)
	THF	393.48(B) 611.40(Q)	1.87 0.08	H-1→L + 1(25.6%) H→L (26.9%)		399.58(B) 569.25(Q)	1.96 0.09	H-1→L (28.6%) H→L (32.1%)
	ETOH	391.94(B) 610.80(Q)	1.81 0.08	H-1→L + 1(25.3%) H→L (27.2%)		398.30 (B) 569.46(Q)	1.91 0.09	H-1→L (28.3%) H→L (32.1%)
**III**	GAS	375.05(B) 619.95(Q)	1.73 0.07	H-1→L (28.5%) H→L (34%)	**VIII**	373.51 (B) 561.29 (Q)	1.66 0.05	H-1→L + 1(16.5%) H→L (28.2%)
	THF	396.06(B) 612.84(Q)	1.91 0.13	H-1→L (27%) H→L (32.7%)		396.43 (B) 568.62 (Q)	1.92 0.09	H-1→L + 1(24%) H→L (31%)
	ETOH	399.25(B) 622.94(Q)	1.89 0.13	H-1→L (25.2) H→L (35.4%)		395.11 (B) 568.91 (Q)	1.88 0.09	H-1→L + 1(23.8%) H→L (31%)
**IV**	GAS	370.63(B) 612.86(Q)	1.64 0.05	H-1→L + 1(22.1%) H→L (22.9%)	**IX**	375.45 (B) 563.90 (Q)	1.65 0.06	H-1→L (26.2%) H→L (31.3%)
	THF	401.63(B) 614.84(Q)	1.88 0.09	H-1→L (25.4%) H→L (26.3%)		399.01 (B) 571.58 (Q)	1.96 0.11	H-1→L (28.8%) H→L (33.3%)
	ETOH	400.9(B) 614.40(Q)	1.81 0.09	H-1→L (24.6%) H→L (26.4%)		397.72 (B) 571.83 (Q)	1.93 0.10	H-1→L (28.6%) H→L (33.3%)
**V**	GAS	370.42(B) 614.32(Q)	1.74 0.05	H-1→L (21.4%) H→L (25.9%)	**X**	373.11 (B) 563.50 (Q)	1.66 0.06	H-1→L (29%) H→L (31.3%)
	THF	395.47(B) 618.29(Q)	1.91 0.11	H-1→L (19.5%) H→L (30.9%)		396.85 (B) 572.31 (Q)	1.93 0.12	H-1→L (31.6%) H→L (33.8%)
	ETOH	394.64(B) 618.19(Q)	1.85 0.11	H-1→L (19.1%) H→L (31.2%)		395.59 (B) 572.76 (Q)	1.89 0.12	H-1→L (31.4%) H→L (33.9%)
**XI**	GAS	384.49(B) 626.53(Q)	0.87 0.22	H-3→L (26.2%) H→L (36.3%)	**XII**	392.83(B) 645.38(Q)	0.83 0.81	H-3→L (22%) H→L (33.8%)
	THF	393.24(B) 629.01(Q)	1.77 0.92	H-2→L + 2(30%) H→L (36%)		394.58(B) 650.9(Q)	1.57 0.90	H-1→L + 2(23.7%) H→L (33.5%)

As demonstrated by Table 4, increasing -NH_2_ donor substitution induces a systematic red-shift in both Soret and Q-band absorption maxima (λ_max_ )Free-base porphyrins (**I–V**) display larger bathochromic shifts than zinc-metalated analogs (**VI–X**), particularly in dyes **III** and **IV** (cis- and trans-di-amino/di-carboxylate configurations). For zinc complexes (**VI–X**), trans-isomer **IX** exhibits the largest red shift ($\:{\lambda\:}_{\mathrm{max}}=571.58\text{ nm}\text{}$in THF). This bathochromic trend is further extended in target dyes **XI** and **XII**, where Q-band absorption reaches 629.01 nm and 650.90 nm in THF, respectively.

Crucially, while Q-band oscillator strengths (* f)* for baseline dyes **I–X** remain small ($\:f\le\:0.13$), target asymmetric dyes **XI** and **XII** exhibit significantly boosted Q-band oscillator strengths of 0.92 and 0.90 in THF, respectively. This intensity enhancement stems from extended donor conjugation breaking macrocyclic symmetry $\:({D}_{4\mathrm{h}}\to\:{C}_{1}\:)$ and intensifying the $\:{S}_{0}\to\:{S}_{1}$transition dipole moment^13,19^.

**Fig. 7:**
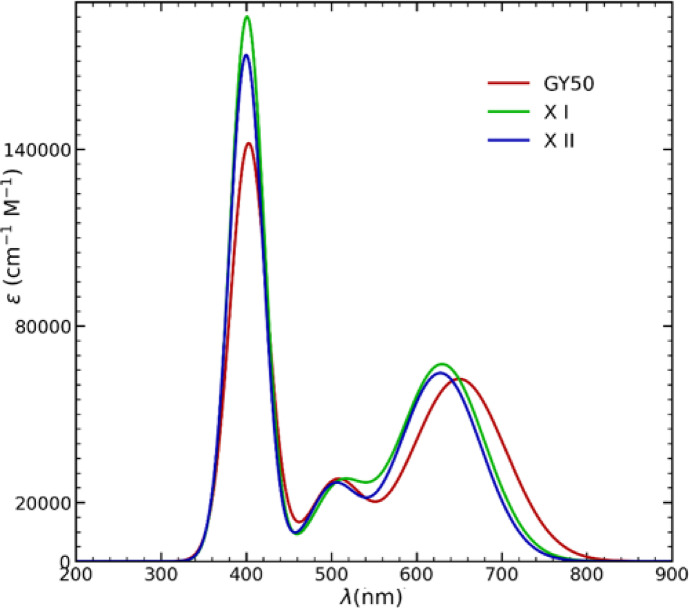
Calculated electronic UV-Vis absorption spectra (ε vs. λ) comparing benchmark zinc-porphyrin **GY50**^33^ with target modeled sensitizers XI and XII in THF solvent (CAM-B3LYP/LANL2DZ/C-PCM), illustrating enhanced Q-band absorption in the 500–700 nm region.

Simulated absorption spectra (Fig. 7) reveal maximum Soret band molar extinction coefficients ($\:{\epsilon\:}_{\mathrm{max}}\:)$reaching $\:1.8\times\:{10}^{5}{\text{ M}}^{-1}{\mathrm{cm}}^{-1}\:$for **XI** and $\:1.7\times\:{10}^{5}{\text{ M}}^{-1}{\mathrm{cm}}^{-1}$ for **XII**, compared to $\:1.4\times\:{10}^{5}{ \text{ M}}^{-1}{\mathrm{cm}}^{-1}\:$for **GY50**^33^. These higher molar extinction coefficients demonstrate superior light-harvesting capacity across the visible spectrum.

To validate these computational spectral predictions against experimental standards, our TD-DFT metrics were compared with published experimental data for the benchmark dye **GY50**^4,33^. In THF, experimental **GY50** exhibits a high-intensity Soret absorption peak at $\:{\lambda\:}_{\mathrm{max}}\approx\:448\text{ nm}$ and a prominent Q-band at $\:{\lambda\:}_{\mathrm{max}}\approx\:648\text{ nm}$^33^. Our CAM-B3LYP calculations yield corresponding absorption peaks at 393.24 nm (Soret) and 629.01 nm (Q-band) for **XI**, and 394.58 nm (Soret) and 650.90 nm (Q-band) for **XII**. This agreement confirms that range-separated TD-DFT successfully reproduces the experimental optical red-shifting trends and Q-band intensity enhancements induced by asymmetric donor modification^28,29,33^.

**Fig. 8:**
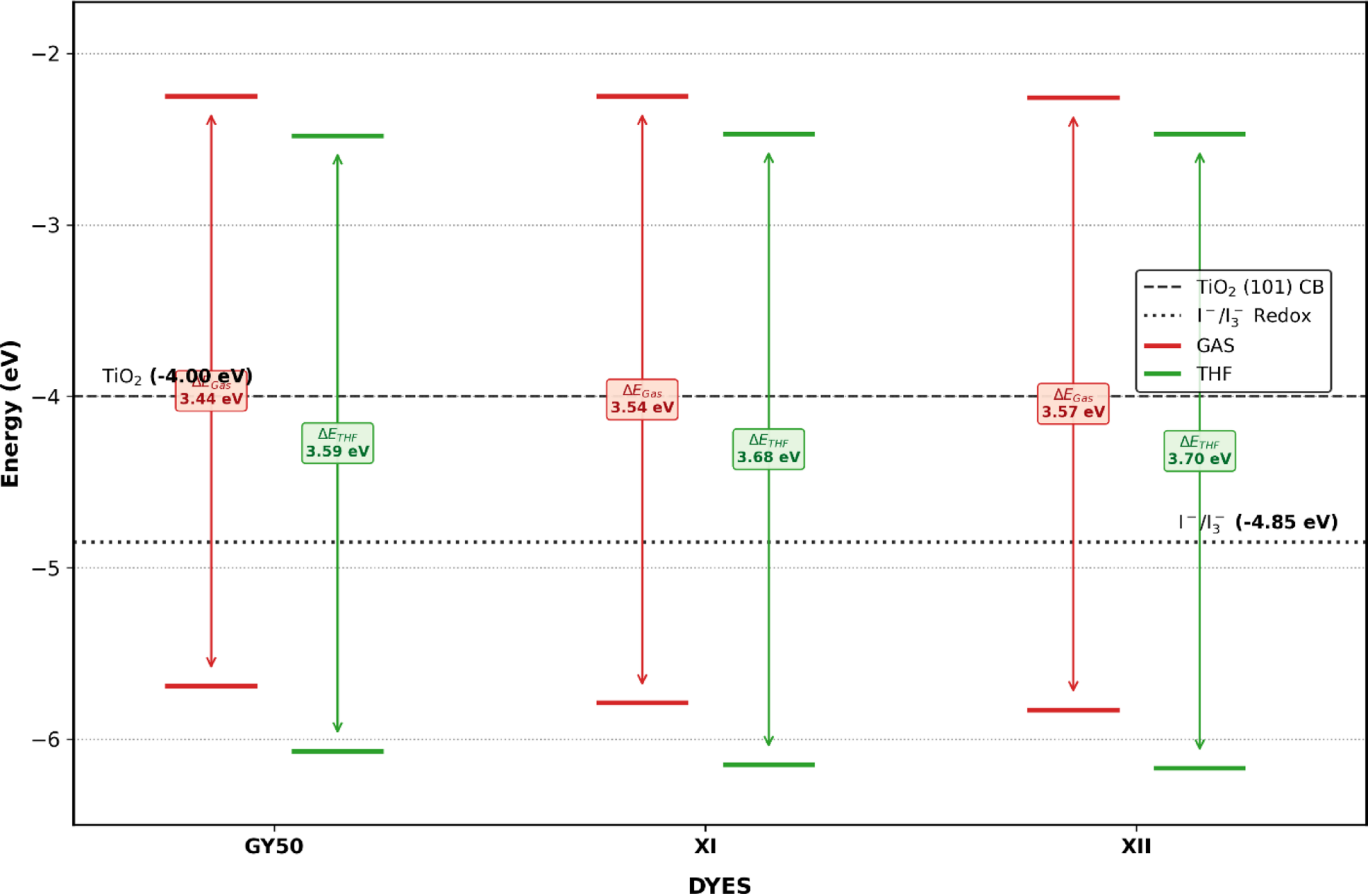
Redesigned schematic energy band alignment diagram illustrating calculated HOMO and LUMO energy levels, explicit HOMO–LUMO energy gaps (E_g_), and thermodynamic driving forces ($\:\varDelta\:{G}_{\mathrm{injection}},\:\varDelta\:{G}_{\mathrm{regeneration}}$) for benchmark Dye **GY50**^33^ and target Dyes XI and XII in gas and THF phases relative to the anatase $\:{\mathrm{TiO}}_{2}\left(101\right)\:$conduction band edge and the $\:{\mathrm{I}}^{-}/{\mathrm{I}}_{3}^{-}$electrolyte redox potential.

Relative frontier orbital alignments dictate the thermodynamic feasibility of interfacial charge separation and sensitizer regeneration. As shown in the redesigned energy alignment diagram (Fig. 8), $\:{E}_{\mathrm{LUMO}}\:$levels for benchmark **GY50** (-2.20 eV) and target models **XI** (-2.46 eV) and **XII** (-2.47 eV) are situated well above the anatase TiO_2_(101) conduction band edge^43,44^. This energetic offset establishes a substantial thermodynamic driving force $\:\varDelta\:{G}_{\mathrm{injection}}=-1.54{\:eV}\text{}$for **XI** and $\:-1.53{\:eV}\text{}$for **XII**) for rapid electron injection into the photoanode. Concurrently, ground-state $\:{E}_{\mathrm{HOMO}}\:$levels $\:-6.14{\:eV}\text{}$for **XI** and $\:-6.17{\:eV}\text{}$for **XII**) lie deeply below the Nernstian redox potential of the $\:{\mathrm{I}}^{-}/{\mathrm{I}}_{3}^{-}\:$electrolyte couple, providing a robust driving force $\:\varDelta\:{G}_{\mathrm{regeneration}}=-1.29{\:eV}\text{}$for **XI** and $\:-1.32{\:eV}\text{}$for **XII**) for prompt dye reduction by the redox medium^44^.

### Photoelectrochemical descriptors and charge-transfer metrics

Computed photoelectrochemical parameters, thermodynamic driving forces, and spatial charge-transfer metrics for dyes **I–XII** are summarized in Table 5. Light-harvesting efficiency (LHE) quantifies the photon absorption probability of the sensitizer layer. For baseline porphyrin models **I–X**, calculated LHE values at the Soret (B) band absorption maximum converge near unity (LHE_B_ = 0.98–0.99), whereas Q-band light-harvesting capacity remains low (LHE_Q_ = 0.07–0.26) due to macrocyclic symmetry constraints^27^.

For target sensitizers **XI** and **XII**, LHE_Q_ increases significantly to 0.88 in THF, matching their high Soret performance (LHE_B_ = 0.97–0.98$). This dual-band absorption profile indicates that photoinduced electron injection in **XI** and **XII** can proceed efficiently from both lower-lying S_1_ (Q-band) and higher-lying S_2_ (B-band) excited states, broadening the effective photo-response window across the visible spectrum^19^.

**Table 5:** Calculated photoelectrochemical descriptors, light-harvesting efficiencies (LHE) thermodynamic driving forces (ΔG), and spatial charge-transfer metrics (q_CT_, d_CT_, t) for dyes **I–XII**.

Dye	Solvent	$\:{\mathrm{LHE}}_{\mathrm{B}}$	$\:{\mathrm{LHE}}_{\boldsymbol{Q}}$		$\:-\boldsymbol{\varDelta\:}{\boldsymbol{G}}_{\mathrm{inj}}^{\mathrm{B}}$(eV)	$\:-\boldsymbol{\varDelta\:}{\boldsymbol{G}}_{\mathrm{inj}}^{\boldsymbol{Q}}\left(\mathbf{e}\mathbf{V}\right)$		$\:\boldsymbol{\varDelta\:}{\boldsymbol{G}}_{\mathrm{reg}}$(eV)	$\:\boldsymbol{\varDelta\:}{\boldsymbol{G}}_{\mathbf{i}\mathbf{n}\mathbf{j}}^{\mathbf{L}\mathbf{U}\mathbf{M}\mathbf{O}}$(eV)	$\:{\boldsymbol{q}}_{\mathrm{CT}}$ (e)	d_CT_ (A˚)	t (A˚)
**I**	Gas	0.99	0.08		0.86	0.47		1.67	1.83	0.17	0.03	3.78
	THF	0.99	0.12		0.62	0.52		1.72	1.78	0.19	0.05	3.84
	EtOH	0.99	0.12		0.63	0.52		1.73	1.77	0.19	0.05	3.84
**II**	Gas	0.98	0.16		1.12	0.19		1.37	2.03	0.19	0.30	3.75
	THF	0.99	0.17		0.79	0.33		1.51	1.86	0.22	0.54	3.69
	EtOH	0.98	0.17		0.78	0.35		1.53	1.83	0.22	0.59	3.67
**III**	Gas	0.98	0.15		1.36	-0.05		1.10	2.23	0.22	0.22	3.92
	THF	0.99	0.26		0.92	0.19		1.36	1.98	0.26	0.25	4.14
	EtOH	0.99	0.26		0.87	0.25		1.39	1.88	0.27	0.51	4.04
**IV**	Gas	0.98	0.11		1.28	0.05		1.22	2.13	0.19	0.09	4.14
	THF	0.99	0.19		0.84	0.23		1.40	1.91	0.23	0.09	4.35
	EtOH	0.99	0.19		0.82	0.26		1.42	1.88	0.23	0.09	4.38
**V**	Gas	0.98	0.12		1.63	-0.30		0.86	2.49	0.21	0.15	4.00
	THF	0.99	0.23		1.05	0.08		1.23	2.07	0.25	0.26	4.20
	EtOH	0.99	0.23		1.01	0.13		1.28	2.00	0.25	0.29	4.23
**VI**	Gas	0.98	0.07		-0.81	0.32		1.70	1.90	0.17	0.05	4.90
	THF	0.99	0.11		0.70	0.25		1.61	1.96	0.19	0.05	4.86
	EtOH	0.99	0.11		0.73	0.24		1.59	1.97	0.19	0.05	4.86
**VII**	Gas	0.98	0.11		1.05	0.04		1.39	2.09	0.16	0.65	4.16
	THF	0.99	0.19		0.84	0.08		1.41	2.03	0.20	0.84	4.12
	EtOH	0.99	0.18		0.85	0.09		1.41	2.02	0.20	0.88	4.10
**VIII**	Gas	0.98	0.11		1.31	-0.20		1.16	2.36	0.17	0.79	3.97
	THF	0.99	0.19		1.00	-0.06		1.28	2.16	0.23	1.07	3.98
	EtOH	0.99	0.19		1.00	-0.04		1.29	2.14	0.21	1.12	3.98
**IX**	Gas	0.98	0.13		1.21	-0.10		1.24	2.20	0.17	0.13	4.69
	THF	0.99	0.22		0.95	-0.01		1.31	2.08	0.21	0.16	4.77
	EtOH	0.99	0.21		0.95	-0.003		1.32	2.07	0.21	0.16	4.78
**X**	Gas	0.98	0.13		1.58	-0.46		0.89	2.56	0.17	0.34	4.38
	THF	0.99	0.24		1.13	-0.17		1.14	2.24	0.22	0.52	4.44
	EtOH	0.99	0.24		1.11	-0.14		1.18	2.20	0.23	0.55	4.45
**XI**	GAS	0.87	0.40		1.44	-0.19		0.94	1.75	0.56	3.08	3.73
	THF	0.98	0.88		1.00	0.17		1.29	1.54	0.48	3.17	2.93
**XII**	GAS	0.85	0.85		1.33	-0.10		0.97	1.74	0.55	2.98	3.72
	THF	0.97	0.88		0.98	0.26		1.32	1.53	0.47	3.10	2.90

Thermodynamic driving forces for electron injection from Soret ($\:-\varDelta\:{G}_{\mathrm{inj}}^{B})$ and Q-band ($\:-\varDelta\:{G}_{\mathrm{inj}}^{Q})$ excited states confirm that interfacial charge transfer is energetically favorable $\:(\varDelta\:G<0$) across the series. For target dyes **XI** and **XII** in THF, LUMO-level injection driving forces ($\:\varDelta\:{G}_{\mathrm{inj}}^{\mathrm{LUMO}})$ converge at 1.54 eV and 1.53 eV, respectively. Concurrently, ground-state dye regeneration driving forces ($\:\varDelta\:{G}_{\mathrm{reg}}$) range from 1.14 eV to 1.73 eV, ensuring rapid reduction of the oxidized dye cation ($\:{\mathrm{dye}}^{+})$ by the electrolyte and suppressing dark-current recombination^44^.

Qualitatively, $\:{V}_{\mathrm{oc}}\:$capability is influenced by the molecular dipole moment component perpendicular to the surface ($\:{\mu\:}_{n}$). As the ratio of -NH_2_ donors to carboxylate anchors increases (moving from **I** to **V** and **VI** to **X**), the positive vertical dipole moment increases, inducing a favorable upward shift in the semiconductor conduction band edge ($\:\varDelta\:{E}_{\mathrm{CB}}$)^43^.

Quantitative charge-transfer analysis executed in Multiwfn^39,40^ demonstrates that net transferred electronic charge (q_CT_ ) averages 0.22 e for baseline dyes **I–X** in THF but increases to 0.48 e for **XI** and 0.47 e for **XII** (Table 5). Concurrently, charge-transfer distance (d_CT_) reaches 3.17 A˚ and 3.10 A˚ for **XI** and **XII**, respectively. These elevated q_CT_ and d_CT_ values confirm that asymmetric donor modification enhances spatial charge separation upon vertical photoexcitation^40^.

### Interfacial reorganization energies and injection kinetics

The interfacial electron injection rate constant ($\:{k}_{\mathrm{injection}})$ from photoexcited sensitizers into the TiO_2_ conduction band matrix is governed by non-adiabatic Marcus electron-transfer theory^45,47,48^. The kinetic barrier for electron transfer is directly mediated by internal molecular reorganization energy ($\:{\lambda\:}_{i}={\lambda\:}_{h}+{\lambda\:}_{e}$), which quantifies structural relaxation during hole ($\:{\lambda\:}_{h}$) and electron ($\:{\lambda\:}_{e})$ transfer processes^45^. A lower $\:{\lambda\:}_{i}$ minimizes structural distortion during transitions, reducing the free energy activation barrier, accelerating $\:{k}_{\mathrm{injection}}$, and mitigating competitive non-radiative decay^45,48^.

Given the macrocyclic structural similarity across series **I–XII**, the electronic coupling pre-factor (A) and outer-sphere solvent reorganization energy ($\:{\lambda\:}_{o}$) can be treated as virtually constant within a given solvent medium. Consequently, evaluating the Marcus exponential factor, $\:\mathrm{exp}\left(-{\lambda\:}_{i}/4{k}_{\mathrm{B}}T\right)\:$at 298.15 K, provides a direct qualitative metric for comparing relative interfacial injection kinetics^45^.

**Table 6:** Calculated Hole (λ_h_), Electron (λ_e_), and Total Internal Reorganization Energies (λ_i_) along with Marcus Exponential Kinetic Transport Factors at 298.15 K.

Dye	λ_h_ (eV)	λ_e_ (eV)	λ_i_ (eV)	$\:\mathbf{exp}\left(-{\boldsymbol{\lambda\:}}_{\boldsymbol{i}}/4{\boldsymbol{k}}_{\mathrm{B}}\boldsymbol{T}\right)$	Dye	λ_h_ (eV)	λ_e_ (eV)	λ_i_ (eV)	$\:\mathbf{exp}\left(-{\boldsymbol{\lambda\:}}_{\boldsymbol{i}}/4{\boldsymbol{k}}_{\mathrm{B}}\boldsymbol{T}\right)$
I	0.121	0.297	0.418	0.017	VI	0.131	0.297	0.428	0.016
II	0.205	0.313	0.518	0.006	VII	0.207	0.278	0.485	0.009
III	0.245	0.260	0.505	0.007	VIII	0.235	0.252	0.487	0.009
IV	0.286	0.329	0.615	0.002	IX	0.290	0.282	0.572	0.004
V	0.318	0.269	0.587	0.003	X	0.325	0.239	0.564	0.004
XI	0.093	0.226	0.319	0.045	XII	0.157	0.220	0.377	0.026

Note: $\:{\lambda\:}_{h}$ and $\:{\lambda\:}_{e}$denote hole and electron reorganization energies, respectively; $\:{\lambda\:}_{i}={\lambda\:}_{h}+{\lambda\:}_{e}$

As compiled in Table 6, baseline free-base (**I–V**) and zinc-complexed (**VI–X**) series display average internal reorganization energies of 0.53 eV and 0.51 eV, respectively. Systems bearing four carboxylates (**I**, **VI**) or symmetrical di-amino/di-carboxylate topologies (**III**, **VIII**) exhibit the lowest baseline kinetic barriers ($\:{\lambda\:}_{i}$ = 0.418–0.505 eV).

Crucially, target sensitizers **XI** and **XII** display exceptionally low internal reorganization energies of 0.319 eV and 0.377 eV, respectively. These minimized reorganization energies translate directly into the largest Marcus kinetic factors in the series: $\:\mathrm{exp}\left(-{\lambda\:}_{i}/4{k}_{\mathrm{B}}T\right)=0.045\:$ (**XI**) and 0.026 (**XII**). This reduction demonstrates that **XI** and **XII** undergo minimal structural deformation upon photoexcitation and oxidation, lowering the activation barrier for charge transfer to accelerate interfacial electron injection into the semiconductor 3d-conduction band matrix^45,48^.

### Surface adsorption dynamics and electronic coupling on TiO_2_(101)

**Fig. 9:**
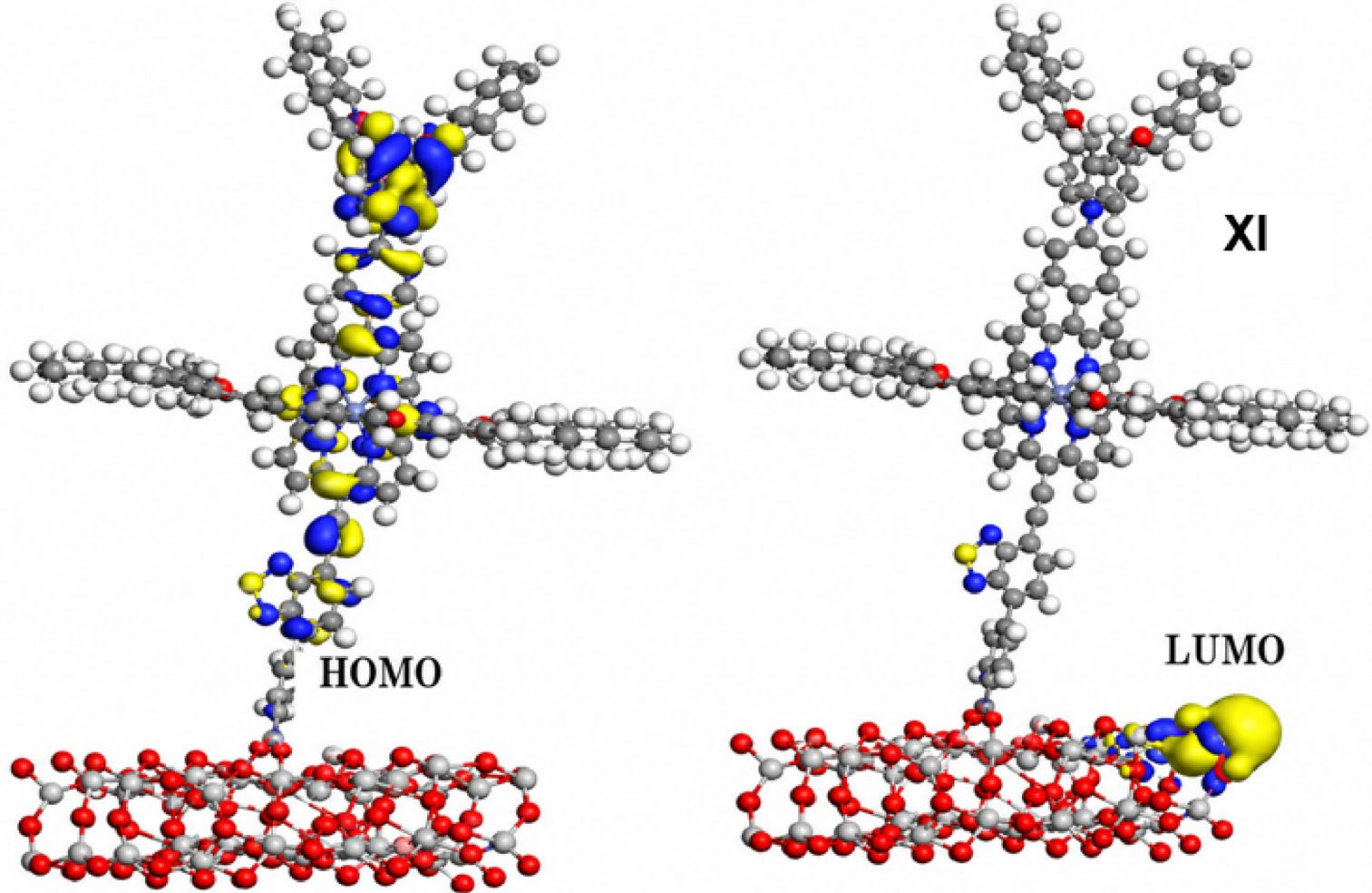
Spatial electronic density distributions of HOMO and LUMO energy levels for target modeled dye **XI** anchored onto the anatase TiO_2_(101) surface via a (TiO_2_)_36_ cluster model, calculated at the GGA/PBE-DNP level of theory.

**Fig. 10:**
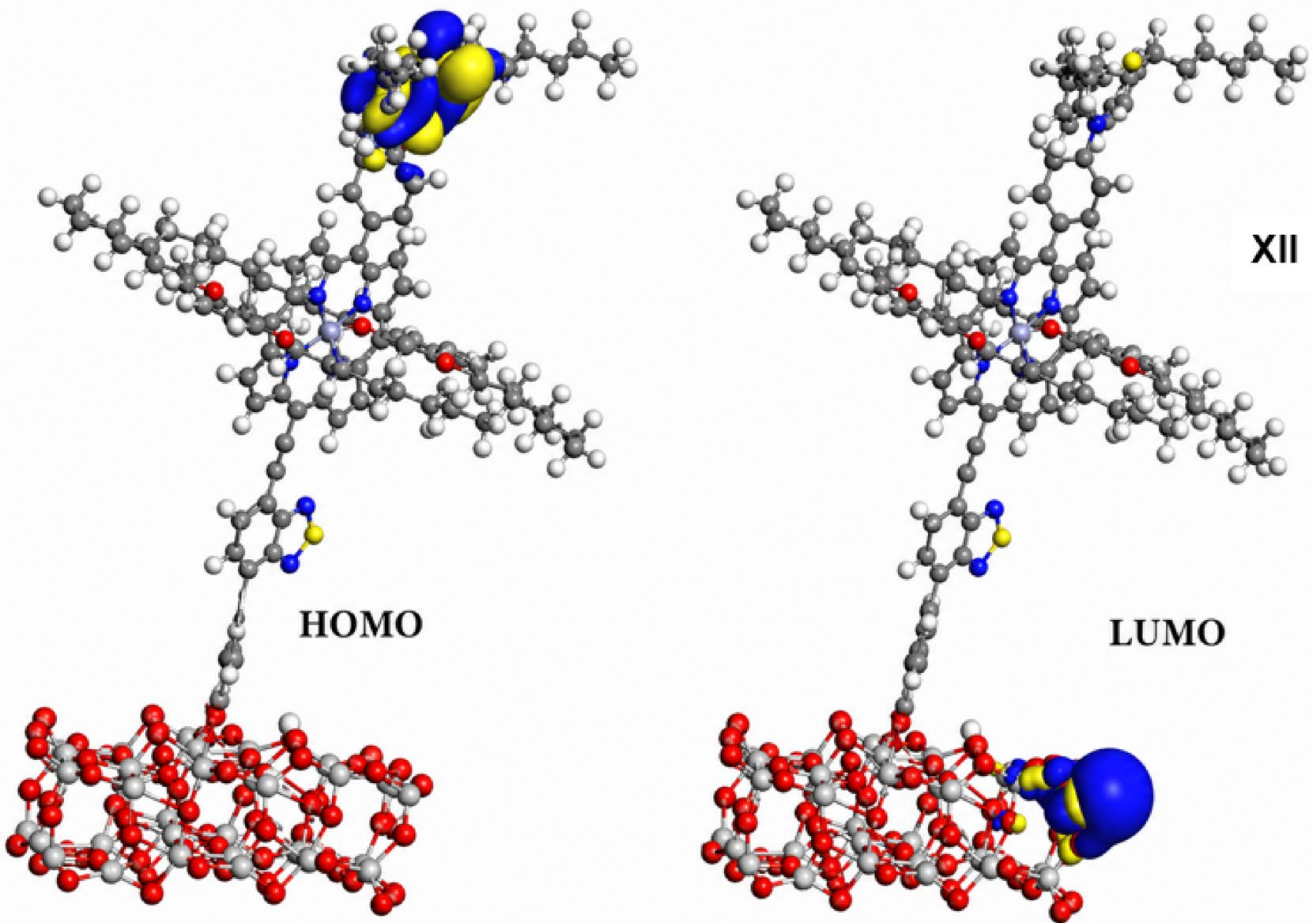
Spatial electronic density distributions of HOMO and LUMO energy levels for target modeled dye **XII** anchored onto the anatase TiO_2_(101) surface via a (TiO_2_)_36_ cluster model, calculated at the GGA/PBE-DNP level of theory.

To model the solid-state photoanode interface, chemisorption configurations, binding energies, and structural parameters of zinc-porphyrins VI–XII anchored onto the dominant (101) facet of anatase $\:{\mathrm{TiO}}_{2}$ were evaluated using a periodic-sized $\:{\left({\mathrm{TiO}}_{2}\right)}_{36}\:$cluster model within DMol³ (GGA/PBE-DNP)^34–37^. The anatase (101) facet constitutes over 90% of exposed surface area in nanostructured $\:{\mathrm{TiO}}_{2}\:$films^35,36,50^. Carboxylic acid (-COOH) groups anchor preferentially via a bidentate bridging coordination mode, wherein one carboxylate oxygen binds to a 5-fold coordinated surface titanium atom ($\:{\mathrm{Ti}}_{5\mathrm{c}}$), while the hydroxyl proton transfers to an adjacent 2-fold coordinated surface oxygen atom ($\:{\mathrm{O}}_{2\mathrm{c}})$^5,35,43^. Optimized interfacial configurations of target dyes **XI** and **XII** on $\:{\left({\mathrm{TiO}}_{2}\right)}_{36}\:$ are depicted in Figs. 9 and 10, respectively.

Calculated negative adsorption energies ($\:\varDelta\:{E}_{\mathrm{ads}})$ confirm that chemisorption onto $\:{\mathrm{TiO}}_{2}\left(101\right)$ is thermodynamically spontaneous and exothermic across all zinc complexes (Table 7). Among baseline complexes, trans-di-amino-di-carboxylate dye **IX** exhibits the strongest binding energy $\:(\varDelta\:{E}_{\mathrm{ads}}=-19.52\text{ kcal/mol}$), surpassing its cis-isomer **VIII** (-16.97 kcal/mol) due to reduced steric congestion at the anchor interface^35^.

**Table 7:** Calculated exothermic adsorption energies ($\:\varDelta\:{E}_{\mathrm{ads}}$) and interfacial surface Ti–O bond lengths for Zinc-Porphyrins **VI–XII** anchored on the anatase $\:{\mathrm{TiO}}_{2}\left(101\right)$cluster (GGA/PBE-DNP).

Dye	ΔE_ads_ (kcal/mol)	Ti–O_1_ (A˚)	Ti–O_2_ (A˚)
**VI**	-1.73	2.095	2.075
**VII**	-10.12	2.093	2.069
**VIII**	-16.97	2.096	2.069
**IX**	-19.52	2.096	2.070
**X**	-16.34	2.097	2.068
**XI**	-15.21	2.068	2.085
**XII**	-16.71	2.086	2.067

Note: The total electronic energy of the clean, relaxed (TiO_2_)_36_ cluster was calculated as $\:{E}_{{\mathrm{TiO}}_{2}}$= -4,719,462.79 kcal/mol. Adsorption energy is defined as $\:\varDelta\:{E}_{\mathrm{ads}}={E}_{{\mathrm{dye+TiO}}_{2}}-\left({E}_{\mathrm{dye}}+{E}_{{\mathrm{TiO}}_{2}}\right)$.

Newly modeled target dyes XI and XII display robust surface chemisorption energies of -15.21 kcal/mol and − 16.71 kcal/mol, respectively. Calculated interfacial bond distances between surface $\:{\mathrm{Ti}}_{5\mathrm{c}}\:$atoms and carboxylate oxygen atoms range tightly between 2.067 A˚ and 2.097 A˚, matching experimental X-ray crystallographic and DFT standards for bidentate chemisorption^35,43,51^.

Furthermore, 3D spatial electron density distributions of anchored systems (Figs. 9 and 10) confirm that unoccupied LUMO wavefunctions of **XI** and **XII** extend across the carboxylate anchoring linkage directly into the empty 3d-conduction band matrix of TiO_2_ This spatial electronic coupling ensures rapid, direct interfacial electron injection upon photoexcitation^35,46^.

Out-of-plane *meso*-phenyl ring rotations effectively suppress face-to-face $\:(\pi\:\mathrm{--}\pi\:$) molecular aggregation at the interface^13,30^. However, it should be noted that the current adsorption model considers an isolated single-molecule monolayer on the $\:{\left({\mathrm{TiO}}_{2}\right)}_{36}$cluster without explicit solvent or multi-dye excitonic dimer interactions, which will be addressed in future interfacial modeling studies.

## Conclusions

Systematic quantum chemical evaluation of free-base (**I–V**) and zinc-complexed (**VI–X**) baseline porphyrins demonstrates that peripheral donor (-NH_2_) and acceptor (-COOH) topology dictates optical harvesting and interfacial energetics. In these baseline systems, photo-response is dominated by Soret-band absorption with primary electron injection originating from the S_2_ state. While *trans*-configured di-amino (**III**, **VIII**, **IX**) and tri-amino (**V**, **X**) architectures enhance Q-band sensitization and optimize energy level alignment, they exhibit moderate spatial charge transfer and substantial internal reorganization energies. Interfacial modeling on $\:{\left({\mathrm{TiO}}_{2}\right)}_{36}\:$clusters confirms that *trans*-isomers minimize steric congestion to yield superior thermodynamic binding affinity, as seen in dye **IX** ($\:\varDelta\:{E}_{\mathrm{ads}}=-19.52{\:kcal/mol}$) compared to its *cis*-analogue **VIII** ($\:\varDelta\:{E}_{\mathrm{ads}}=-16.97{\:kcal/mol}$).

Symmetry breaking ($\:{D}_{4\mathrm{h}}\to\:{C}_{1}$) combined with asymmetric donor extension in target sensitizers **XI** and **XII** directly resolves these baseline limitations. This architectural modification dramatically intensifies Q-band oscillator strength (* f* = 0.92), elevating its light-harvesting efficiency (LHE_Q_ = 0.88) to match the Soret band (LHE_B_ = 0.98) and enabling efficient dual-band photon capture across both S_1_ and S_2_ excited states. Simultaneously, spatial charge transfer increases significantly to $\:{q}_{\mathrm{CT}}=0.48\hspace{0.17em}e\:$over an intramolecular distance of $\:{d}_{\mathrm{CT}}=3.17{\:A^\circ}$.

Crucially, dyes **XI** and **XII** exhibit exceptionally low reorganization energies $\:{(\lambda\:}_{i}=0.319{\:eV}\text{}$and 0.377 eV), suppressing nuclear relaxation barriers to maximize the Marcus kinetic transport factor ($\:\mathrm{exp}\left[-{\lambda\:}_{i}/4{k}_{\mathrm{B}}T\right])$ and accelerate interfacial electron injection. On anatase TiO_2_(101), both target dyes maintain stable bidentate chemisorption ($\:\varDelta\:{E}_{\mathrm{ads}}$ = -15.21 kcal/mol and − 16.71 kcal/mol), while out-of-plane *meso*-phenyl rings effectively suppress π-π molecular aggregation.

While these findings establish a strong predictive framework, the current model is subject to computational limitations, including reliance on truncated surface cluster models, single-molecule monolayer assumptions, and the absence of explicit interfacial solvent molecules. Future work will focus on wet-chemical synthesis of dyes **XI** and **XII**, photoanode fabrication, co-sensitization strategies to extend panchromatic harvesting into the near-infrared regime, and multi-dye modeling to evaluate explicit intermolecular excitonic interactions.

## Supplementary Information

Below is the link to the electronic supplementary material.

Supplementary Material 1

## Data Availability

“All data generated or analysed during this study are included in this published article”.
