# Supplementary material for: Advancing dye sensitized solar cell efficiency and computational insights into porphyrin based dyes
Source: Sci Rep. 2026 Aug 17;16:25722. doi: 10.1038/s41598-026-66026-x (PMC13482394; doi:10.1038/s41598-026-66026-x)
Supplement: Supplementary file 1 [file 41598_2026_66026_MOESM1_ESM.docx]

**Advancing Dye Sensitized Solar Cell Efficiency and Computational Insights into Porphyrin Based Dyes**

Noha Khamis, Yasser R. Elmarassi, Ahmed A. Hasanein, Howida A. Fetouh^*^

^a^ Chemistry Department, Faculty of Science, Alexandria University, Egypt

[*howida_fetouh@alexu.edu.eg](mailto:*howida_fetouh@alexu.edu.eg)

**Fig.S.1:** Representation of HOMO-1, HOMO, LUMO, and LUMO+1 for iso-surfaces of dyes I and II obtained by B3LYP/LANL2DZ method

**Fig.S.2**: Representation of HOMO and LUMO energy levels for the adsorbed dye I on anatase (101) surface, calculated by PBE/DNP in gas phase

**Table S1:** Comparative Energetic and Reactivity Indices for Dyes VI–X calculated using B3LYP/LANL2DZ Level

| Dye | Medium | E_Total_ (au) | μ  (D) | E_HOMO_ | E_LUMO_ | HOMO-LUMO gap | ∆E _solvation_  (kcal/mol) |
| --- | --- | --- | --- | --- | --- | --- | --- |
| **VI** | GAS | -2732.05 | 1.68 | -5.62 | -2.92 | 2.70 | - |
|  | THF | -2732.09 | 2.12 | -5.52 | -2.85 | 2.67 | -26.01 |
|  | ETOH | -2732.09 | 2.19 | -5.50 | -2.83 | 2.67 | -29.08 |
| **VII** | GAS | -2598.86 | 5.06 | -5.28 | -2.72 | 2.56 | - |
|  | THF | -2598.90 | 5.70 | -5.27 | -2.77 | 2.50 | -26.06 |
|  | ETOH | -2598.90 | 5.77 | -5.26 | -2.78 | 2.48 | -29.12 |
| **VIII** | GAS | -2465.66 | 9.03 | -5.01 | -2.44 | 2.57 | - |
|  | THF | -2465.70 | 10.39 | -5.12 | -2.63 | 2.49 | -25.22 |
|  | ETOH | -2465.70 | 10.57 | -5.14 | -2.65 | 2.49 | -28.20 |
| **XI** | GAS | -2465.65 | 4.21 | -5.11 | -2.60 | 2.51 | - |
|  | THF | -2465.69 | 4.55 | -5.15 | -2.71 | 2.44 | -28.57 |
|  | ETOH | -2465.70 | 4.59 | -5.15 | -2.72 | 2.43 | -32.00 |
| **X** | GAS | -2332.46 | 6.23 | -4.76 | -2.22 | 2.54 | - |
|  | THF | -2332.50 | 7.15 | -4.99 | -2.54 | 2.45 | -25.14 |
|  | ETOH | -2332.51 | 7.26 | -5.02 | -2.58 | 2.44 | -28.14 |
|  |  |  |  |  |  |  |  |
